# Reassessing Simultaneous Pancreas-Kidney Vs. Kidney Transplant Alone: A Propensity-Weighted Analysis of Survival and Morbidity

**DOI:** 10.3389/ti.2025.14934

**Published:** 2025-12-30

**Authors:** Pooja Budhiraja, Rocio Lopez, Susana Arrigain, Jesse D. Schold

**Affiliations:** 1 Department of Medicine, Mayo Clinic Arizona, Phoenix, AZ, United States; 2 Department of Surgery, University of Colorado Anschutz Medical Campus, Aurora, CO, United States; 3 Department of Epidemiology, University of Colorado Anschutz Medical Campus, Aurora, CO, United States

**Keywords:** allocation system, SPKT, DDKT, patient survival, allograf survival

## Abstract

This study compares outcomes between Simultaneous Pancreas-Kidney Transplantation (SPKT) and Deceased Donor Kidney Transplantation (DDKT) in recipients with diabetes, assessing survival benefits against surgical and immunological risks. We analyzed Scientific Registry of Transplant Recipients data (2014–2023) to assess patient and kidney graft survival. Overlap propensity score weighting was applied to adjust for group differences. Kaplan-Meier and Cox proportional hazards models were used to estimate survival outcomes in unadjusted, covariate-adjusted, and weighted analyses. Among 22,545 recipients with diabetes (25% SPKT), those receiving SPKT were younger (41 vs. 52 years), predominantly non-Hispanic white, had type 1 diabetes, lower BMI, shorter dialysis duration, and higher preemptive transplant rates (all p < 0.001). Overlap-weighted (ow) analyses showed no significant differences in 5- and 10-year patient (SPKT: 86%, 71%; DDKT: 87%, 74%) and kidney graft survival (SPKT: 80%, 66%; DDKT: 83%, 62%). SPKT recipients with graft survival at 1 year experienced higher 1-year treated acute rejection (owOR: 2.80, 95% CI: 1.75–4.49) and hospital readmissions (owOR: 2.05, 95% CI: 1.62–2.60). However, among recipients with type 1 diabetes and BMI <30, SPKT was associated with lower mortality compared to DDKT. After adjustment for selection bias, SPKT did not improve long-term survival compared to DDKT and was associated with greater early morbidity.

## Graphical Abstract

**Figure:**
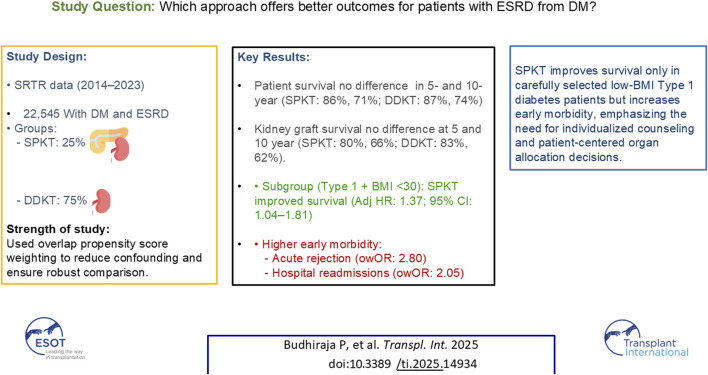

## Introduction

Kidney transplantation is a standard therapeutic intervention for chronic and end-stage renal disease (ESRD). In select patients with diabetes and kidney failure, a simultaneous pancreas and kidney transplant (SPKT) also restores euglycemia. It normalizes glycosylated hemoglobin levels, further improving quality of life and reducing diabetic complications in this select recipient candidate population [1–3].

Despite its many benefits, SPKT carries notable challenges, including a 5%–10% increased risk of early pancreas graft loss, higher early postoperative complications, a greater rate of early hospital readmissions, and a higher incidence of combined graft rejection compared to kidney-alone transplants [4].

Previous studies comparing SPKT with Deceased Donor Kidney Transplantation (DDKT) have suggested potential survival and metabolic advantages of SPKT, particularly among recipient with type 1 diabetes. SPKT is associated with a lower incidence of major adverse cardiovascular events, enhanced left ventricular function, and improved metabolic control associated with euglycemia, which are critical for patients with diabetes and end-stage renal disease [5–7]. Recent literature highlights the protective effects of SPKT on cardiac function and vascular health, likely due to the restoration of insulin production and improved glycemic control [8, 9]. Furthermore, SPKT recipients experience improved quality of life metrics and metabolic control, which help delay or reverse diabetic complications and improve long-term survival [5–7]. Additionally, there is growing consideration for expanding SPKT criteria to patients with type 2 diabetes, reflecting evolving clinical practice trends [10].

Despite these benefits, SPKT is associated with higher surgical morbidity, increased early complications, and higher acute rejection rates. Survival rates in successful SPKT recipients are reportedly higher when the pancreas functions effectively, yet the trade-off between risk and benefit remains debatable [11, 12].

Previous studies assessing outcomes after SPKT primarily evaluated highly selected cohorts, specifically patients with type 1 or type 2 diabetes, separately, highlighting significant survival benefits associated with early pancreas graft function. However, such analyses did not fully account for critical donor and recipient selection biases or incorporate comprehensive adjustments for minimal overlap scenarios between SPKT and DDKT cohorts [1]. Moreover, most earlier studies did not robustly quantify surgical morbidity, including acute rejection rates and hospital readmissions, which may have led to an incomplete picture of overall clinical benefits.

The comparative benefits of SPKT versus DDKT remain challenging to assess due to inherent differences in recipient selection practices, variability in donor organ quality, and the trade-offs between surgical and immunological risks versus potential metabolic improvements and quality-of-life gains from pancreas transplantation. Our study uniquely addresses these critical gaps by employing advanced overlap propensity score weighting techniques designed to mitigate selection bias across a broader recipient cohort with diabetes, rigorously evaluating multiple outcome measures beyond pancreas graft function alone and reflecting contemporary surgical practices and immunosuppression protocols. By precisely matching SPKT and DDKT recipients on critical donor and recipient characteristics, we provide a more accurate comparative assessment of the true benefits and risks associated with SPKT.

Assessing the risks versus benefits of SPKT is essential for informed patient counseling. It weighs surgical risks against metabolic control and quality of life, helping patients choose between SPKT and DDKT. This study aims to illuminate these considerations, thereby improving patient guidance and optimizing organ allocation policies to maximize the advantages of these critical transplants.

## Materials and Methods

This study used data from the Scientific Registry of Transplant Recipients (SRTR). The SRTR data system includes data on all donor, wait-listed candidates, and transplant recipients in the US, submitted by the members of the Organ Procurement and Transplantation Network (OPTN). The Health Resources and Services Administration (HRSA), U.S. Department of Health and Human Services provides oversight to the activities of the OPTN and SRTR contractors. We used the September 2024 standard analysis files to identify subjects who received kidney or simultaneous kidney-pancreas transplantation between 1 January 2014, and 30 November 2023. The following exclusions were applied: recipients younger than 18 or older than 59 years at time of transplant, non-diabetic recipient, primary diagnosis other than diabetes, prior kidney or pancreas transplant, multi-organ transplant other than kidney-pancreas, living donor, en-bloc or sequential kidney transplant, donor younger than 18 or older than 59 (Supplementary Figure S1).

Our primary outcomes were time to kidney allograft failure and patient death. We defined kidney allograft survival time as the number of months from transplantation to irreversible graft failure, indicated by a return to dialysis, kidney re-transplantation, or patient death [13]. We censored at the earliest of the recipient censoring cohort date or the last graft follow-up date. For our cohort, we defined patient survival as the number of months from transplantation until death or the recipient censoring cohort date, which was 1 June 2024. We truncated follow-up at 10 years.

Secondary outcomes included risk for treated acute kidney graft rejection either prior to discharge or during the first year post-transplant. Rejection events reflect treated acute kidney rejection. Biopsy confirmation is not consistently performed or coded across centers in the registry, so we relied on treatment-based indicators. Therefore, we chose to use acute treated kidney rejection as a practical alternative. While this may slightly overstate rejection incidence, treated episodes are likely to reflect clinically significant cases, minimizing overestimation.

We also assessed hospital readmission during the first year post-transplant. To ensure equal time risk when examining outcomes in the first-year post-transplant, we restricted the analysis to the subset of subjects with a 1-year post-transplant follow-up form who had not experienced graft loss within the first post-transplant year. Additionally, we examined 90-day pancreas graft failure among the SKPT group, defined as the number of days from transplantation to irreversible graft failure, as indicated by documented graft failure, pancreas re-transplantation, or death. Additionally, we calculated the Pancreas Donor Risk Index (PDRI) using the formula presented by Axelrod et al. [14].

We calculated the Kidney Donor Profile Index (KDPI) using the 2014 reference values, as this was the midpoint of our cohort [15].

Data were missing for the following variables: education (1.4%), donor history of hypertension (1.2%), donor history of diabetes (1.1%), KDPI (0.74%), BMI (0.72%), donor eGFR (0.71%), cold ischemia time (0.49%), donor BMI (0.29%), dialysis duration at transplant (0.16%), peak cPRA (0.13%), pancreas procedure type (0.04%), donor race/ethnicity (0.02%), and primary insurance (0.009%).

Continuous variables were summarized using means and standard deviations (SD), and categorical factors were summarized using frequencies and percentages. We used t-tests and Pearson’s chi-square tests to compare continuous and categorical variables between the DDKT and SPKT groups. Post-transplant length of stay was summarized with median, 25th, and 75th percentiles and compared with Wilcoxon rank sum tests due to its skewed distribution.

We used multivariate imputation by chained equations to impute 5 datasets with complete data. The multiple imputation included the following characteristics: age, sex, race/ethnicity, BMI, education, insurance, dialysis duration, insurance, diabetes type, time of on the waitlist, dialysis duration, cPRA, cold ischemia time, donor sex, donor age, donor race/ethnicity, donor BMI, donor history of diabetes, donor history of hypertension, KDPI, deceased donor type, donor cause of death, transplant type, kidney transplant type, graft loss, and graft survival time. All models were fitted on each of the 5 imputed datasets, and parameter estimates were combined.

We utilized overlap propensity score weighting to address potential confounding factors arising from the significant differences in recipient and donor characteristics between DDKT and SPKT. To estimate the propensity score for receiving a SPKT, we employed a multivariable logistic regression model that included recipient age, sex, race/ethnicity, BMI, education, insurance, dialysis duration, diabetes type, blood type, time of on the waitlist, cPRA, cold ischemia time, left vs. right kidney transplant, donor sex, donor age, donor race/ethnicity, donor BMI, donor history of diabetes, donor history of hypertension, donor blood type, KDPI, deceased donor type (DBD vs. DCD), donor cause of death, and transplant year. Supplementary Figure S2 illustrates the distributions of propensity scores based on transplant type. The overlap propensity score weighting method assigns each patient’s weight based on the probability of that patient receiving the alternative transplant type [16] and has been shown to outperform inverse probability of treatment weighting (IPTW) in cases of minimal overlap [17].

We plotted unweighted and overlap-weighted Kaplan-Meier estimates to visualize the cumulative rates of kidney allograft failure and patient mortality. Additionally, we employed Cox proportional hazards models to assess three scenarios: 1) the unadjusted association, 2) the covariate-adjusted association (using the same variables as the propensity score model), and 3) the overlap-weighted association between transplant type and the cumulative outcomes of kidney allograft failure and patient mortality. We examined the interaction between transplant type and age group in covariate-adjusted and overlap-weighted models.

We utilized logistic regression to evaluate the association between transplant type and treated acute rejection and hospital readmissions. These models were built under the same three scenarios as the Cox models using the subset of subjects with the 1-year post-transplant follow-up form who had not experienced graft loss within the first post-transplant year. We also calculated the cumulative 90-day pancreas graft survival in the SKPT group and used a log-rank test to evaluate differences by age group.

Lastly, we conducted sensitivity analyses restricted to recipients with Type 1 diabetes to assess the robustness of our findings in a subgroup more closely aligned with SPK listing criteria. We performed two subgroup analyses: 1) recipients with Type 1 diabetes, and 2) recipients with Type 1 diabetes and BMI <30, comparing outcomes for DDKT versus SPKT.

All tests were two-tailed and performed at a significance level of 0.05. Analyses were performed using SAS 9.4 software (SAS Institute, Cary, NC).

## Results

**FIGURE 1:**
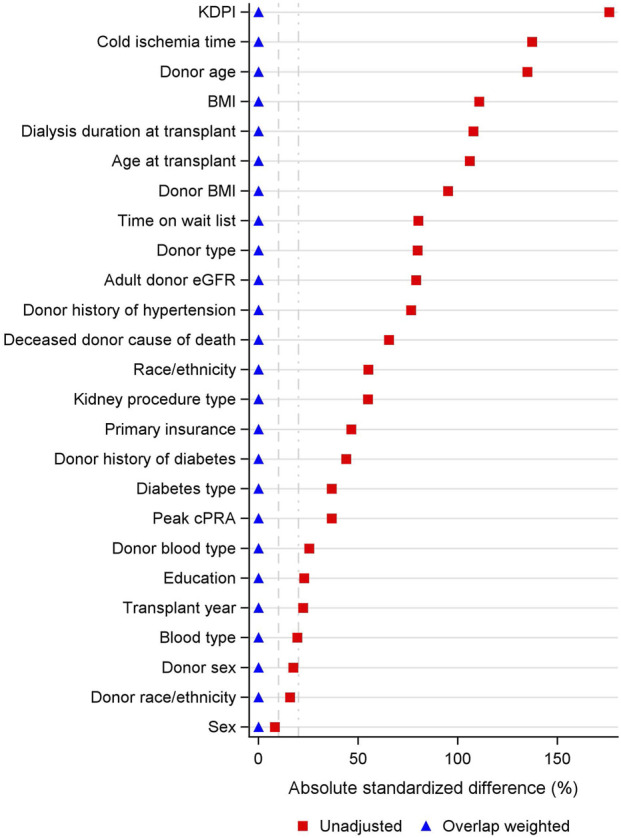
Covariate Balance Across SKPT vs. DDKT. BMI: body mass index; cPRA: calculated panel reactive antibody; eGFR: estimated glomerular filtration rate; KDPI: kidney donor profile index.

This analysis included 22,545 transplant recipients with diabetes, 25% of whom received a SPKT. SPKT recipients were younger than DDKT recipients (mean ± SD: 42 ± 9 vs. 50 ± 7 years, p < 0.001) (Table 1). Additionally, a higher percentage of SPKT recipients were female (39% vs. 35%, p = 0.003) and non-Hispanic white (48% vs. 23%, p < 0.001). Regarding insurance, 51% of SPKT and 71% of DDKT recipients had Medicare. SPKT recipients had a lower BMI (26 ± 4 vs. 31 ± 5 kg/m^2^, p < 0.001) and were more likely to have type 1 diabetes (79% vs. 12%, p < 0.001) than DDKT recipients. SPKT recipients had a higher rate of preemptive transplants (15% vs. 4% in DDKT, p < 0.001), shorter durations of dialysis, and waitlist time (52% vs. 30% in the <5-month category, p < 0.001). Due to the large sample size, many comparisons reached statistical significance. The absolute standard differences between the groups can be seen in Figure 1.

**TABLE 1:** Recipient and procedure characteristics by transplant type.

Factor	Overall (N = 22,545)		DDKT (N = 16,793)		SPKT (N = 5,752)		p-value
	N missing	Statistics	N missing	Statistics	N missing	Statistics	
Age at transplant (years)	0	48.2 ± 8.4	0	50.4 ± 7.0	0	41.6 ± 8.6	*<0.001* ^a^
Age at transplant (years)	0	​	0	​	0	​	***<0.001*** ^***c***^
18–39	​	4,058 (18.0)	​	1,530 (9.1)	​	2,528 (43.9)	​
40–49	​	6,782 (30.1)	​	4,793 (28.5)	​	1,989 (34.6)	​
50–59	​	11,705 (51.9)	​	10,470 (62.3)	​	1,235 (21.5)	​
Sex	0	​	0	​	0	​	***<0.001*** ^***c***^
Female	​	8,077 (35.8)	​	5,847 (34.8)	​	2,230 (38.8)	​
Male	​	14,468 (64.2)	​	10,946 (65.2)	​	3,522 (61.2)	​
Race/ethnicity	0	​	0	​	0	​	***<0.001*** ^***c***^
Non-hispanic white	​	6,639 (29.4)	​	3,870 (23.0)	​	2,769 (48.1)	​
Non-hispanic black	​	8,500 (37.7)	​	6,793 (40.5)	​	1,707 (29.7)	​
Non-hispanic other and Multi-racial	​	1,992 (8.8)	​	1,711 (10.2)	​	281 (4.9)	​
Hispanic	​	5,414 (24.0)	​	4,419 (26.3)	​	995 (17.3)	​
Education	312	​	216	​	96	​	***<0.001*** ^***c***^
High school or less	​	11,102 (49.9)	​	8,711 (52.5)	​	2,391 (42.3)	​
Some college	​	6,321 (28.4)	​	4,552 (27.5)	​	1,769 (31.3)	​
College or more	​	4,810 (21.6)	​	3,314 (20.0)	​	1,496 (26.4)	​
Primary insurance	2	​	0	​	2	​	***<0.001*** ^***c***^
Private	​	5,662 (25.1)	​	3,387 (20.2)	​	2,275 (39.6)	​
Medicare	​	14,886 (66.0)	​	11,925 (71.0)	​	2,961 (51.5)	​
Medicaid	​	1,587 (7.0)	​	1,146 (6.8)	​	441 (7.7)	​
Other	​	408 (1.8)	​	335 (2.0)	​	73 (1.3)	​
BMI	163	29.5 ± 5.4	140	30.8 ± 5.2	23	25.8 ± 4.0	***<0.001*** ^***a***^
BMI	163	​	140	​	23	​	***<0.001*** ^***c***^
<18.5	​	172 (0.77)	​	71 (0.43)	​	101 (1.8)	​
18.5–24.9	​	4,784 (21.4)	​	2,323 (13.9)	​	2,461 (43.0)	​
25–29.9	​	7,343 (32.8)	​	5,005 (30.1)	​	2,338 (40.8)	​
30–34.9	​	6,359 (28.4)	​	5,591 (33.6)	​	768 (13.4)	​
≥35	​	3,724 (16.6)	​	3,663 (22.0)	​	61 (1.06)	​
Diabetes type	0	​	0	​	0	​	***<0.001*** ^***c***^
Type I	​	6,512 (28.9)	​	1,959 (11.7)	​	4,553 (79.2)	​
Type II	​	16,033 (71.1)	​	14,834 (88.3)	​	1,199 (20.8)	​
Blood type	0	​	0	​	0	​	***<0.001*** ^***c***^
A	​	8,016 (35.6)	​	6,060 (36.1)	​	1,956 (34.0)	​
AB	​	1,330 (5.9)	​	1,106 (6.6)	​	224 (3.9)	​
B	​	3,188 (14.1)	​	2,476 (14.7)	​	712 (12.4)	​
O	​	10,011 (44.4)	​	7,151 (42.6)	​	2,860 (49.7)	​
Dialysis duration at transplant (months)	37	​	21	​	16	​	***<0.001*** ^***c***^
Preemptive	​	1,586 (7.0)	​	704 (4.2)	​	882 (15.4)	​
>0–11.9	​	2,307 (10.2)	​	1,156 (6.9)	​	1,151 (20.1)	​
12–23.9	​	3,129 (13.9)	​	1,625 (9.7)	​	1,504 (26.2)	​
24–47.9	​	5,512 (24.5)	​	4,059 (24.2)	​	1,453 (25.3)	​
48–71.9	​	4,767 (21.2)	​	4,276 (25.5)	​	491 (8.6)	​
≥72	​	5,207 (23.1)	​	4,952 (29.5)	​	255 (4.4)	​
Peak cPRA	30	​	29	​	1	​	***<0.001*** ^***c***^
0	​	13,130 (58.3)	​	9,272 (55.3)	​	3,858 (67.1)	​
1–19	​	2,604 (11.6)	​	1,918 (11.4)	​	686 (11.9)	​
20–79	​	4,250 (18.9)	​	3,314 (19.8)	​	936 (16.3)	​
80–97	​	1,528 (6.8)	​	1,294 (7.7)	​	234 (4.1)	​
97–100	​	1,003 (4.5)	​	966 (5.8)	​	37 (0.64)	​
Transplant year	0	​	0	​	0	​	***<0.001*** ^***c***^
2014	​	1,646 (7.3)	​	1,143 (6.8)	​	503 (8.7)	​
2015	​	1,593 (7.1)	​	1,065 (6.3)	​	528 (9.2)	​
2016	​	1,840 (8.2)	​	1,283 (7.6)	​	557 (9.7)	​
2017	​	1,958 (8.7)	​	1,410 (8.4)	​	548 (9.5)	​
2018	​	2,096 (9.3)	​	1,498 (8.9)	​	598 (10.4)	​
2019	​	2,450 (10.9 )	​	1,805 (10.7)	​	645 (11.2)	​
2020	​	2,571 (11.4)	​	1,973 (11.7)	​	598 (10.4)	​
2021	​	2,800 (12.4)	​	2,187 (13.0)	​	613 (10.7)	​
2022	​	2,899 (12.9)	​	2,294 (13.7)	​	605 (10.5)	​
2023	​	2,692 (11.9)	​	2,135 (12.7)	​	557 (9.7)	​
Kidney procedure type	0	​	0	​	0	​	***<0.001*** ^***c***^
Left kidney	​	12,339 (54.7)	​	8,082 (48.1)	​	4,257 (74.0)	​
Right kidney	​	10,206 (45.3)	​	8,711 (51.9)	​	1,495 (26.0)	​
Time on wait list (months)	0	​	0	​	0	​	***<0.001*** ^***c***^
0–5.9	​	7,969 (35.3)	​	4,994 (29.7)	​	2,975 (51.7)	​
6–11.9	​	3,055 (13.6)	​	1,983 (11.8)	​	1,072 (18.6)	​
12–23.9	​	3,665 (16.3)	​	2,643 (15.7)	​	1,022 (17.8)	​
24–47.9	​	4,206 (18.7)	​	3,664 (21.8)	​	542 (9.4)	​
≥48	​	3,650 (16.2)	​	3,509 (20.9)	​	141 (2.5)	​
Cold ischemia time (hours)	111	​	48	​	63	​	***<0.001*** ^***c***^
<6	​	1,306 (5.8)	​	586 (3.5)	​	720 (12.7)	​
6–11.9	​	5,763 (25.7)	​	2,584 (15.4)	​	3,179 (55.9)	​
12.0–23.9	​	11,269 (50.2)	​	9,528 (56.9)	​	1,741 (30.6)	​
≥24.0	​	4,096 (18.3)	​	4,047 (24.2)	​	49 (0.86)	​

Statistics presented as Mean ± SD or N (column %).p-values: a = t-test, c = Pearson’s chi-square test.

BMI: Body mass index; cPRA: calculated panel reactive antibody; DDKT: deceased donor kidney transplant; SPKT: simultaneous pancreas-kidney transplant.

Bold values denote statistically significant results at the prespecified significance level (P < 0.05).

The cold ischemia time was shorter for SPKT than DDKT (69% vs. 19% in the <12 h category, p < 0.001). Notably, 74% of SPKT recipients received a left kidney compared to 48% of DDKT recipients.

SPKT donors were younger than DDKT donors (27 ± 7 vs. 41 ± 11 years, p < 0.001) (Table 2). Additionally, a higher percentage of SPKT donors were male (70% vs. 62%, p = 0.003) and non-Hispanic Black (20% vs. 15%, p < 0.001). SPKT donors were also less likely to have a history of diabetes or hypertension and had significantly higher eGFR. Additionally, SPKT individuals were more likely to be DBD and had lower KDPI.

**TABLE 2:** Donor characteristics by transplant type.

Factor	Overall (N = 22,545)		DDKT (N = 16,793)		SPKT (N = 5,752)		p-value
	N missing	Statistics	N missing	Statistics	N missing	Statistics	
Donor age (years)	0	37.4 ± 11.6	0	41.0 ± 10.7	0	26.8 ± 6.9	*<0.001* ^a^
Donor age (years)	0	​	0	​	0	​	***<0.001*** ^***c***^
18–39	​	12,878 (57.1)	​	7,418 (44.2)	​	5,460 (94.9)	​
40–49	​	5,269 (23.4)	​	5,000 (29.8)	​	269 (4.7)	​
50–59	​	4,398 (19.5)	​	4,375 (26.1)	​	23 (0.40)	​
Donor sex	0	​	0	​	0	​	***<0.001*** ^***c***^
Female	​	8,069 (35.8)	​	6,363 (37.9)	​	1,706 (29.7)	​
Male	​	14,476 (64.2)	​	10,430 (62.1)	​	4,046 (70.3)	​
Donor race/ethnicity	5	​	5	​	0	​	***<0.001*** ^***c***^
Non-hispanic white	​	14,630 (64.9)	​	11,155 (66.4)	​	3,475 (60.4)	​
Non-hispanic black	​	3,629 (16.1)	​	2,491 (14.8)	​	1,138 (19.8)	​
Non-hispanic other and Multi-racial	​	828 (3.7)	​	639 (3.8)	​	189 (3.3)	​
Hispanic	​	3,453 (15.3)	​	2,503 (14.9)	​	950 (16.5)	​
Donor blood type	0	​	0	​	0	​	***<0.001*** ^***c***^
A	​	8,617 (38.2)	​	6,539 (38.9)	​	2,078 (36.1)	​
AB	​	902 (4.0)	​	819 (4.9)	​	83 (1.4)	​
B	​	2,690 (11.9)	​	1,970 (11.7)	​	720 (12.5)	​
O	​	10,336 (45.8)	​	7,465 (44.5)	​	2,871 (49.9)	​
Donor BMI	65	28.0 ± 6.7	65	29.3 ± 7.1	0	24.2 ± 3.7	*<0.001* ^a^
Donor BMI	65	​	65	​	0	​	***<0.001*** ^***c***^
<18.5	​	541 (2.4)	​	300 (1.8)	​	241 (4.2)	​
18.5–24.9	​	7,962 (35.4)	​	4,690 (28.0)	​	3,272 (56.9)	​
25–29.9	​	7,057 (31.4)	​	5,173 (30.9)	​	1,884 (32.8)	​
30–34.9	​	3,831 (17.0)	​	3,511 (21.0)	​	320 (5.6)	​
≥35	​	3,089 (13.7)	​	3,054 (18.3)	​	35 (0.61)	​
Donor history of diabetes	253	1,493 (6.7)	224	1,489 (9.0)	29	4 (0.07)	***<0.001*** ^***c***^
Donor history of hypertension	267	5,625 (25.2)	235	5,360 (32.4)	32	265 (4.6)	***<0.001*** ^***c***^
Donor eGFR (CKD-EPI 2021)	159	​	151	​	8	​	***<0.001*** ^***c***^
120+	​	5,135 (22.9)	​	2,610 (15.7)	​	2,525 (44.0)	​
105–119	​	4,417 (19.7)	​	3,476 (20.9)	​	941 (16.4)	​
90–104	​	2,570 (11.5)	​	1,846 (11.1)	​	724 (12.6)	​
75–89	​	2,422 (10.8)	​	1,802 (10.8)	​	620 (10.8)	​
60–74	​	2,279 (10.2)	​	1,795 (10.8)	​	484 (8.4)	​
≥60	​	5,563 (24.9)	​	5,113 (30.7)	​	450 (7.8)	​
Donor type	0	​	0	​	0	​	***<0.001*** ^***c***^
DBD	​	17,239 (76.5)	​	11,654 (69.4)	​	5,585 (97.1)	​
DCD	​	5,306 (23.5)	​	5,139 (30.6)	​	167 (2.9)	​
Deceased donor cause of death	0	​	0	​	0	​	***<0.001*** ^***c***^
Anoxia	​	10,137 (45.0)	​	8,164 (48.6)	​	1,973 (34.3)	​
Cerebrovascular/Stroke	​	4,544 (20.2)	​	3,973 (23.7)	​	571 (9.9)	​
Head trauma	​	7,105 (31.5)	​	4,037 (24.0)	​	3,068 (53.3)	​
Other	​	759 (3.4)	​	619 (3.7)	​	140 (2.4)	​
KDPI	166	​	157	​	9	​	***<0.001*** ^***c***^
0–19	​	6,037 (27.0)	​	1,963 (11.8)	​	4,074 (70.9)	​
20–39	​	6,888 (30.8)	​	5,532 (33.3)	​	1,356 (23.6)	​
40–59	​	5,481 (24.5)	​	5,198 (31.2)	​	283 (4.9)	​
60–79	​	3,973 (17.8)	​	3,943 (23.7)	​	30 (0.52)	​

Statistics presented as Mean ± SD or N (column %). p-values: a = t-test, c = Pearson’s chi-square test.

BMI: Body mass index; cPRA: calculated panel reactive antibody; DDKT: deceased donor kidney transplant; eGFR: Estimated glomerular filtration rate; KDPI: kidney donor profile index; SPKT: simultaneous pancreas-kidney transplant.

Bold values denote statistically significant results at the prespecified significance level (P < 0.05).

The median post-transplant hospital stay was 8 days [P25, P75: 6, 11] for SPKT recipients compared to 5 days [P25, P75: 4, 7] for DDKT recipients (p < 0.001).

Figure 1 illustrates the covariate balance before and after overlap weighting. The propensity score was estimated using logistic regression, allowing overlap weighting to create exact balance on the mean of every measured covariate. Supplementary Figure S3 displays the relative contribution of each covariate to the propensity score model. Variables with the strongest influence on treatment assignment included recipient blood type, BMI, dialysis duration, and diabetes. Donor characteristics such as blood type, BMI, KDPI, and cold ischemia time were also among the most imbalanced between groups and contributed substantially to the propensity score model to ensure covariate balance, even though they do not directly influence treatment assignment.

**FIGURE 2:**
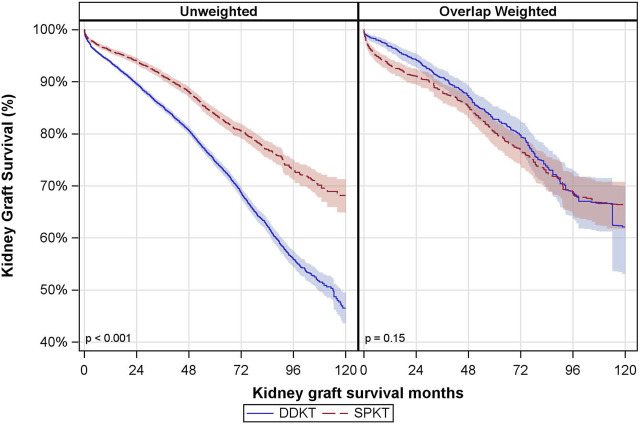
Unadjusted and Overlap Weighted Cumulative Kidney Graft Survival Rates. DDKT: deceased donor kidney transplant; SPKT: simultaneous pancreas-kidney transplant.

*Kidney Graft Survival* The median follow-up time for kidney graft survival was 36 months (P25, P75: 12, 60 months). There was a notable difference in the unadjusted kidney graft survival between the groups, but this did not remain significant after overlap weighting (Figure 2). In the DDKT group, the overlap weighted 5- and 10-year graft survival rates were 83% and 62%, respectively. Compared to the SPKT group, the overlap weighted graft survival rates were 80% at 5 years and 66% at 10 years.

In covariate-adjusted and overlap weighted analyses, there was no significant difference between SPKT vs. DDKT recipients in terms of kidney graft failure (overlap weighted hazard ratio (owHR: 0.86; 95% CI: 0.66, 1.11) (Table 3). The interaction between transplant type and age group was not statistically significant. Results were consistent when looking at death-censored kidney graft failure.

**TABLE 3:** The association of DDKT vs. SPKT with patient mortality and kidney graft failure.

Model	DDKT vs. SPKT HR (95% CI)	
	Kidney graft failure	Patient mortality
Unadjusted	1.76 (1.63, 1.90)	2.06 (1.88, 2.26)
Covariate-adjusted^a^	1.03 (0.91, 1.17)	1.07 (0.92, 1.24)
Overlap weighted	0.86 (0.66, 1.11)	0.85 (0.64, 1.13)

^a^ Adjusted for recipient sex, age, race/ethnicity, education, insurance, blood type, BMI, dialysis months; cPRA, diabetes type, transplant year, left vs. right kidney transplant, months on wait list, CIT, donor sex, donor age, and KDPI.

CI: confidence interval; DDKT: deceased donor kidney transplant; HR: hazard ratio; SPKT: simultaneous pancreas-kidney transplant.

Findings remained consistent in the subset of recipients with Type 1 diabetes, both across all BMI ranges and among those with BMI <30 (Supplementary Table S1).

### Patient Survival

**FIGURE 3:**
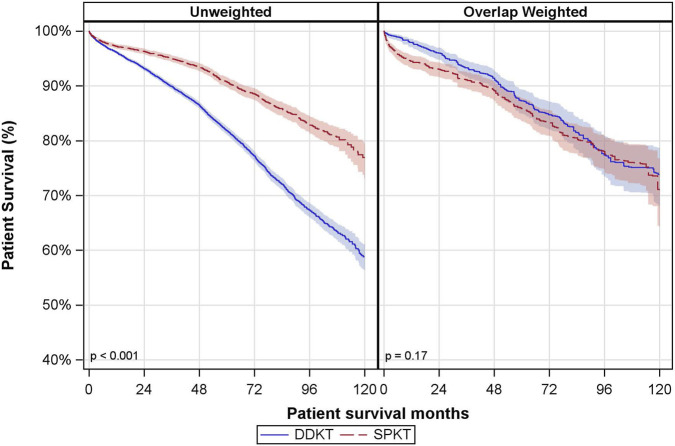
Unadjusted and Overlap Weighted Cumulative Patient Survival Rates. DDKT: deceased donor kidney transplant; SPKT: simultaneous pancreas-kidney transplant.

The median follow-up time for patient survival was 46 months (P25, P75: 24, 72 months). There was a notable difference in the unadjusted patient survival between the groups, but this did not remain significant after overlap weighting (Figure 3). In the DDKT group, the overlap-weighted 5- and 10-year patient survival rates were 87% and 74%, respectively. Comparatively, the SPKT group had overlap-weighted patient survival rates of 86% at 5 years and 71% at 10 years.

In covariate-adjusted and overlap weighted analyses, there was no significant difference in patient mortality between SPKT vs. DDKT recipients (owHR: 0.85; 95% CI: 0.64, 1.13) (Table 3). The interaction between transplant type and age group was not statistically significant.

In the subset of recipients with Type 1 diabetes across all BMI ranges, findings remained consistent with the main analysis. However, among recipients with Type 1 diabetes and BMI <30, those who received DDKT had a significantly higher hazard of death compared to SPKT recipients (adjusted HR: 1.37; 95% CI: 1.04, 1.81) (Supplementary Table S1).

### Treated Acute Rejection and Hospital Readmission

16,793 DDKT and 5,750 SPKT recipients had information regarding treated acute rejection before hospital discharge. Treated acute rejection was documented for 0.78% of each group.

In covariate-adjusted and overlap weighted analyses, there was no significant difference between SPKT and DDKT recipients in terms of treated acute rejection before discharge (overlap weighted odds ratio (owOR): 1.49; 95% CI: 0.32, 6.94) (Table 4).

**TABLE 4:** The association of SPKT vs. DDKT with treated acute rejection and hospital readmission.

Model	SPKT vs. DDKT OR (95% CI)		
	Treated acute Rejection prior to discharge (N = 22,543)	Acute treated Rejection within 1 Year of transplant^a^ (N = 19,072)	Hospital Readmission Within 1 Year of transplant^a^ (N = 19,009)
Unadjusted	1.00 (0.71, 1.41)	2.35 (2.09, 2.64)	1.45 (1.36, 1.55)
Covariate-adjusted^b^	1.51 (0.82, 2.79)	2.96 (2.35, 3.72)	1.77 (1.58, 1.99)
Overlap weighted	1.49 (0.32, 6.94)	2.80 (1.75, 4.49)	2.05 (1.62, 2.60)

^a^ Restricted to the subset of subjects with the 1-year post-transplant follow-up form who had not experienced graft loss within the first post-transplant year.

^b^ Adjusted for sex, age, race/ethnicity, education, insurance, blood type, BMI, dialysis months; cPRA, diabetes type, transplant year, kidney transplant type, months on wait list, CIT, donor sex, donor age, and KDPI.

CI: confidence interval; DDKT: deceased donor kidney transplant; OR: odds ratio; SPKT: simultaneous pancreas-kidney transplant.

A total of 14,107 DDKT and 4,965 SPKT recipients who were alive with a functioning graft 1-year post-transplant had data regarding treated acute rejection within the first year following transplant. Treated acute rejection was documented for 11% of SPKT and only 5% of DDKT. In the covariate-adjusted and overlap-weighted analyses, SPKT recipients were significantly more likely to experience treated acute rejection within 1 year of transplantation than DDKT recipients (owOR: 2.80, 95% CI: 1.75, 4.49) (Table 4).

A total of 14,066 DDKT and 4,943 SPKT recipients who were alive with a functioning graft 1-year post-transplant had data regarding hospital readmissions within a year of transplant. At least one hospital readmission was documented for 62% of SPKT and 52% of DDKT. In the covariate-adjusted and overlap weighted analyses, SPKT recipients were significantly more likely to have hospital readmissions within 1 year of a transplant than DDKT recipients (owOR: 2.05, 95% CI: 1.62, 2.60) (Table 4).

The findings for treated acute rejection and hospital readmission during the first year post-transplant were consistent in the subset of recipients with Type 1 diabetes who remained alive with a functioning graft 1 year after transplantation, across all BMI ranges as well as among those with a BMI less than 30 (Supplementary Table S2). Even among recipients with Type 1 diabetes and BMI under 30, SPKT was associated with an increased risk of treated acute rejection (adjusted OR: 2.08; 95% CI: 1.33–3.25) and hospital readmissions (adjusted OR: 1.62; 95% CI: 1.28–2.04) during the first year following transplant (Supplementary Table S2).

### Pancreas Graft Failure Among SPKT Recipients

Overall, cumulative 90-day pancreas graft survival in the SPKT group was 93% (95% CI: 92, 94), with those aged 50–59 having higher pancreas graft failure rates (Supplementary Figure S4).

## Discussion

The ongoing discussion about the survival benefits of SPKT versus DDKT is inherently complex, compounded by differences in the selectivity of recipient candidates, donor organ quality, and the trade-offs between surgical and immunological risks versus metabolic and quality-of-life benefits of pancreas surgery [1–3, 18]. Additionally, there is growing consideration for expanding SPKT criteria to patients with type 2 diabetes, reflecting evolving clinical practice trends [10].

Historical analyses, particularly using SRTR data, have often indicated superior kidney graft and patient survival rates for SPKT recipients, which were typically attributed to the younger, healthier donor kidneys with lower KDPI scores [4, 11, 12, 19, 20]. Moreover, SPKT’s survival benefits hinge on avoiding early pancreas graft loss [12]. There is a noted risk of 5%–10% for such losses [4]. Also, a pancreas transplant has a higher risk of other surgical complications, such as thrombosis, infections, and leaks, and a higher risk of rejection [21–23].

A significant strength of our study was the robust statistical approach, which employed advanced overlap propensity score weighting, enabling precise balancing of SPKT and DDKT recipients on crucial donor and recipient characteristics, such as donor age, health conditions, and KDPI scores. This meticulous method has been shown to outperform IPTW in cases with minimal overlap [17] and significantly mitigated confounding due to selection bias, providing a more accurate comparative assessment of SPKT outcomes.

Our analysis also highlights key demographic and clinical differences between SPKT and DDKT recipients that may influence transplant outcomes, including insurance coverage, dialysis duration, and racial distribution. SPKT recipients were more likely to have private insurance, shorter dialysis exposure, and to be non-Hispanic white. Among these, shorter dialysis duration prior to transplant is particularly relevant, as it is strongly associated with improved post-transplant survival. Given the large sample size, even small differences between groups may reach statistical significance, and should be interpreted with consideration of their clinical relevance. These findings reinforce the importance of early referral and timely evaluation for transplantation, especially in patients with diabetes, who face high mortality rates while on the waitlist [22].

Although the main analysis demonstrated no significant overall survival benefit for SPKT compared with DDKT, our sensitivity analyses revealed important differences in specific patient subgroups. Among recipients with Type 1 diabetes and BMI <30, SPKT was associated with significantly lower mortality compared to DDKT. This suggests that the survival benefits of SPKT may be most evident in carefully selected, lower-risk patients who meet traditional listing criteria. In contrast, among broader groups that included higher BMI patients or mixed diabetes types, no survival difference was observed. These findings highlight the critical role of patient selection in determining which individuals are most likely to benefit from SPKT.

While this subgroup experienced a survival benefit, SPKT was also associated with higher early morbidity, including treated acute rejection and hospital readmissions, highlighting the need to weigh these risks during transplant decision-making.

SPKT is a more complex surgical procedure than kidney-alone transplantation, with longer operative times and a greater risk of perioperative complications such as thrombosis, infection, and technical graft failures. These early risks may offset the potential survival advantage expected from shorter waitlist times by contributing to higher early postoperative morbidity and mortality. In our study, we observed that 1-year post-transplant outcomes, including treated acute rejection and hospital readmissions, were more frequent among SPKT recipients. This suggests that the clinical burden during the first year after transplant is greater for SPKT recipients, which may further limit the survival benefit of shorter wait time.

An important novel finding in our study was the significantly increased morbidity among SPKT recipients, evidenced by higher rates of treated acute rejection and hospital readmissions within the first post-transplant year among those who were alive with a functioning graft 1-year post-transplant. Highlighting this increased morbidity, despite a lack of survival advantage, underscores the necessity for cautious patient selection and counseling when considering SPKT.

These findings emphasize the trade-off between potential long-term metabolic and survival benefits and the higher early morbidity associated with SPKT. They underscore the importance of individualized counseling and shared decision-making when selecting candidates for SPKT, ensuring that patients understand both the risks and potential advantages.mes.

This study presents several limitations. Firstly, notable differences in recipient characteristics were observed, potentially introducing selection bias in the preference for DDKT over SPKT among candidates with higher surgical risks. The study’s retrospective nature allows for identifying correlations without establishing causality. Additionally, other recipient characteristics not available in the registry may have also impacted the study results. We employed propensity score weighting to mitigate potential confounding due to the notable differences in characteristics between DDKT and SPKT recipients. However, achieving covariate balance on the mean through overlap weighting may not ensure complete adjustment for confounding across all variables. While propensity score weighting reduces bias from measured covariates, it cannot address unmeasured confounders, which may still influence treatment assignment and outcomes, leaving the potential for residual confounding.

Although biopsy-proven rejection offers greater diagnostic accuracy, it is not consistently performed or reliably documented across transplant centers. Therefore, we used treated acute rejection as a practical alternative, reflecting clinically significant episodes while minimizing overestimation. Additionally, the study’s secondary outcomes may not have been consistently captured; however, it is less likely that they were captured in a biased systematic manner between the study groups. Furthermore, these secondary outcomes are assessed using the 1-year follow-up form, and transplant centers are not required to continue follow-up after graft failure. As a result, our analysis is restricted to recipients with 1-year graft survival, which may introduce survivor bias.

The study’s scope was constrained by the limitations of the SRTR database, particularly its inability to monitor diabetic complications such as retinopathy and neuropathy, which are critical to quality of life. Additionally, we lack information regarding post-transplant cardiovascular events and metabolic improvements, both of which are increasingly recognized as important benefits of SPKT over DDKT in recent studies. Recent research has highlighted the advantages of SPKT in reducing cardiovascular morbidity and improving long-term metabolic control. Future research focusing on these areas could yield valuable insights. Furthermore, with the advent of new treatments such as Glucagon-Like Peptide-1 receptor agonists, which have demonstrated cardioprotective and metabolic benefits in population with diabetes, it remains unclear how these therapies may influence outcomes in SPKT versus DDKT recipients. Future research should consider the potential impact of these medications on transplant outcomes, as they could alter the risk-benefit profile of SPKT in managing diabetes-related complications. Prospective studies focusing on these areas could yield valuable insights into the full impact of SPKT on quality of life and long-term survival.

The potential for improved quality of life and long-term metabolic benefits with SPKT is a crucial consideration for patient counseling, particularly for individuals seeking to avoid the costs and burdens associated with insulin therapy. To date, there is a gap in research comparing the cost-effectiveness and quality of life between new insulin therapies and the insulin-free lifestyle afforded by SPKT for patients with diabetes and renal failure. Given the data, the choice of SPKT should be shared with the patient, weighing personal preference against the risk of morbidities versus the potential for increased life years in selected patients.

Our study evaluated transplant data extending through 2023, reflecting contemporary practices in surgical techniques, immunosuppression regimens, and postoperative care. This potentially explains differences in outcomes compared to older studies ending earlier. Over recent years, advances in surgical expertise and immunosuppressive management likely contributed to improved outcomes in kidney-alone transplants, diminishing previously seen advantages of simultaneous pancreas transplants.

Future research should prospectively evaluate diabetic complications and patient-reported outcomes post-transplantation, areas insufficiently captured by registry-based studies. Further cost-effectiveness analyses comparing SPKT with contemporary insulin therapies and newer anti-diabetic medications are essential for comprehensive patient counseling and policymaking.

Our findings emphasize the necessity of individualized patient counseling that comprehensively weighs the risks of increased morbidity against the potential metabolic and quality-of-life benefits of SPKT. Ultimately, these insights necessitate careful reconsideration of existing prioritization policies for SPKT to adopt a nuanced, individualized approach to organ allocation. Ensuring equitable and clinically effective transplant strategies will require balancing demonstrated risks with patient-specific potential benefits. Individualized approaches are needed to balance early surgical risks with potential long-term metabolic and quality-of-life benefits, ensuring that SPKT is prioritized for those most likely to benefit.

## Conclusion

This study adds important context to current organ allocation practices that prioritize SPKT based on presumed survival benefits. Our findings show that, after rigorous statistical adjustment, SPKT recipients face significantly higher early morbidity without clear long-term survival or graft advantages compared to DDKT.

Given these outcomes, organ allocation policies should shift toward individualized approaches, carefully balancing each patient’s clinical risks against potential metabolic and quality-of-life improvements. Patient counseling must reflect these considerations, facilitating informed decisions aligned with patient-specific benefits and risks. Future allocation strategies should also integrate ongoing advancements in diabetes management and address disparities in transplant outcomes across diverse patient populations.

## Data Availability

The original contributions presented in the study are included in the article/Supplementary Material, further inquiries can be directed to the corresponding author.
