# Supplementary material for: Reassessing Simultaneous Pancreas-Kidney Vs. Kidney Transplant Alone: A Propensity-Weighted Analysis of Survival and Morbidity
Source: Transpl Int. 2025 Dec 30;38:14934. doi: 10.3389/ti.2025.14934 (PMC12797420; doi:10.3389/ti.2025.14934)
Supplement: Supplementary file 1 [file Supplementaryfile1.docx]

**Supplemental Material**

**Reassessing Simultaneous Pancreas-Kidney vs Kidney Transplant alone: A Propensity-Weighted Analysis of Survival and Morbidity**

Pooja Budhiraja, Rocio Lopez, Susana Arrigain, Jesse D Schold

Table of Contents

[**Supplemental Figure 1. Patient Selection Flow Chart** 2](#_Toc209171895)

[**Supplemental Figure 2. Propensity Score Distribution** 3](#_Toc209171896)

[**Supplemental Figure 3. Relative Importance of Variables in Propensity Score Estimation** 4](#_Toc209171897)

[**Supplemental Figure 4. Unadjusted Cumulative Pancreas Graft Failure Rates by Age Group** 5](#_Toc209171898)

[**Supplemental Table 1. The association of DDKT vs. SPKT with patient mortality and kidney graft failure, A sensitivity analysis of patients with Type 1 diabetes** 6](#_Toc209171899)

[**Supplemental Table 2. The association of SPKT vs. DDKT with treated acute rejection and hospital readmission, A sensitivity analysis of patients with Type 1 diabetes** 7](#_Toc209171900)

**Supplemental Figure 1. Patient Selection Flow Chart**


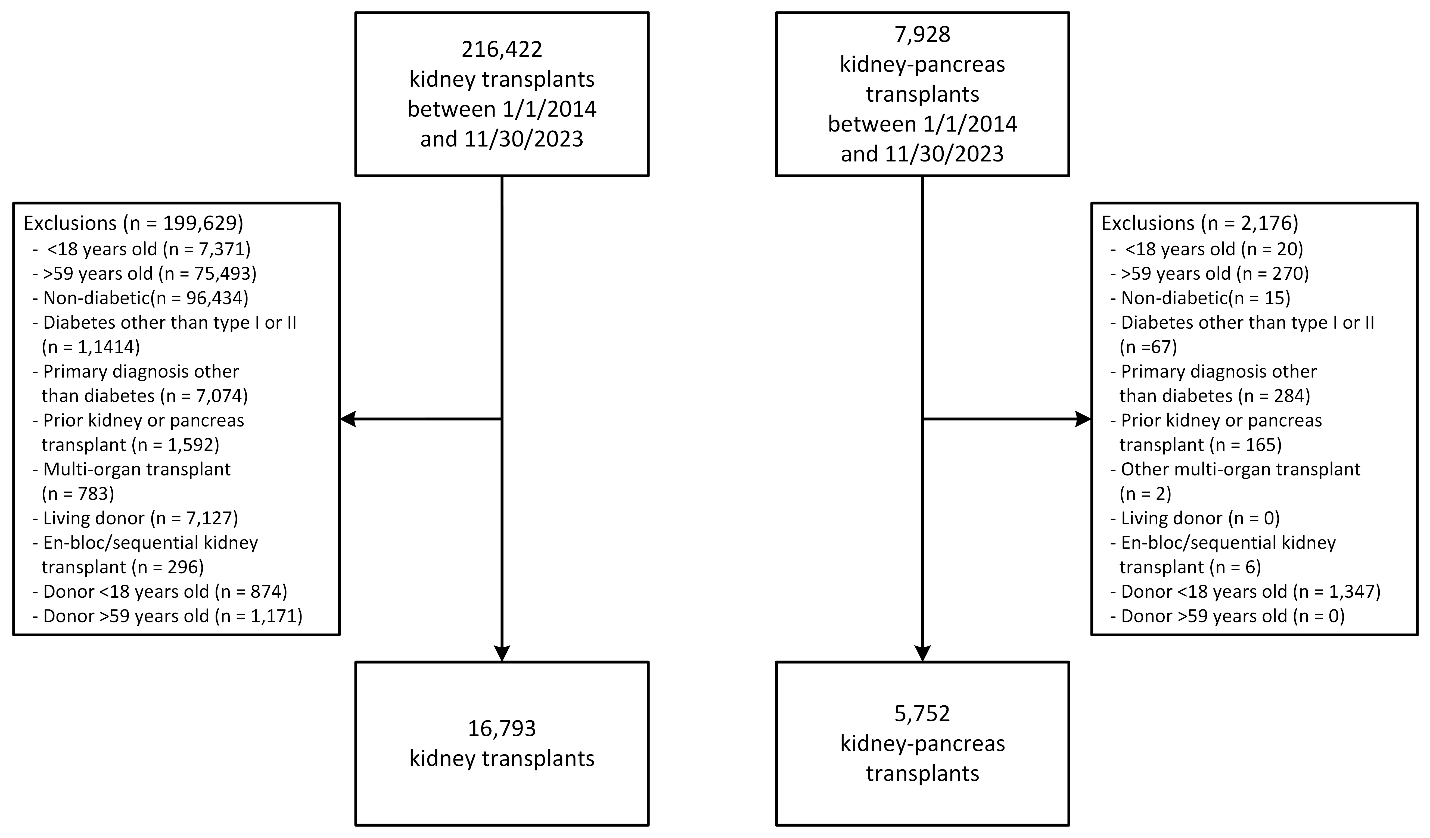


**Supplemental Figure 2. Propensity Score Distribution**


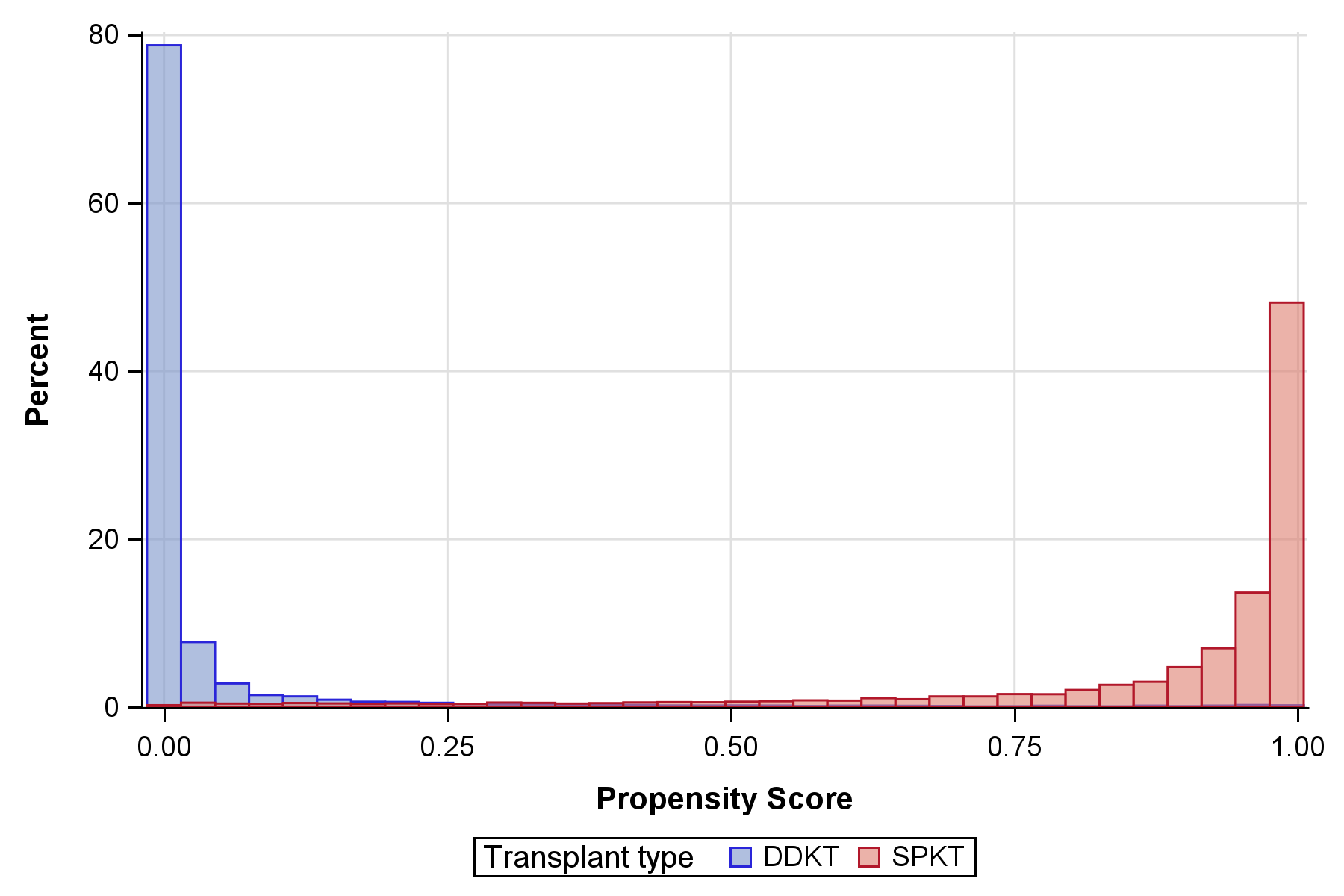


DDKT: deceased donor kidney transplant; SPKT: simultaneous pancreas-kidney transplant

**Supplemental Figure 3. Relative Importance of Variables in Propensity Score Estimation**


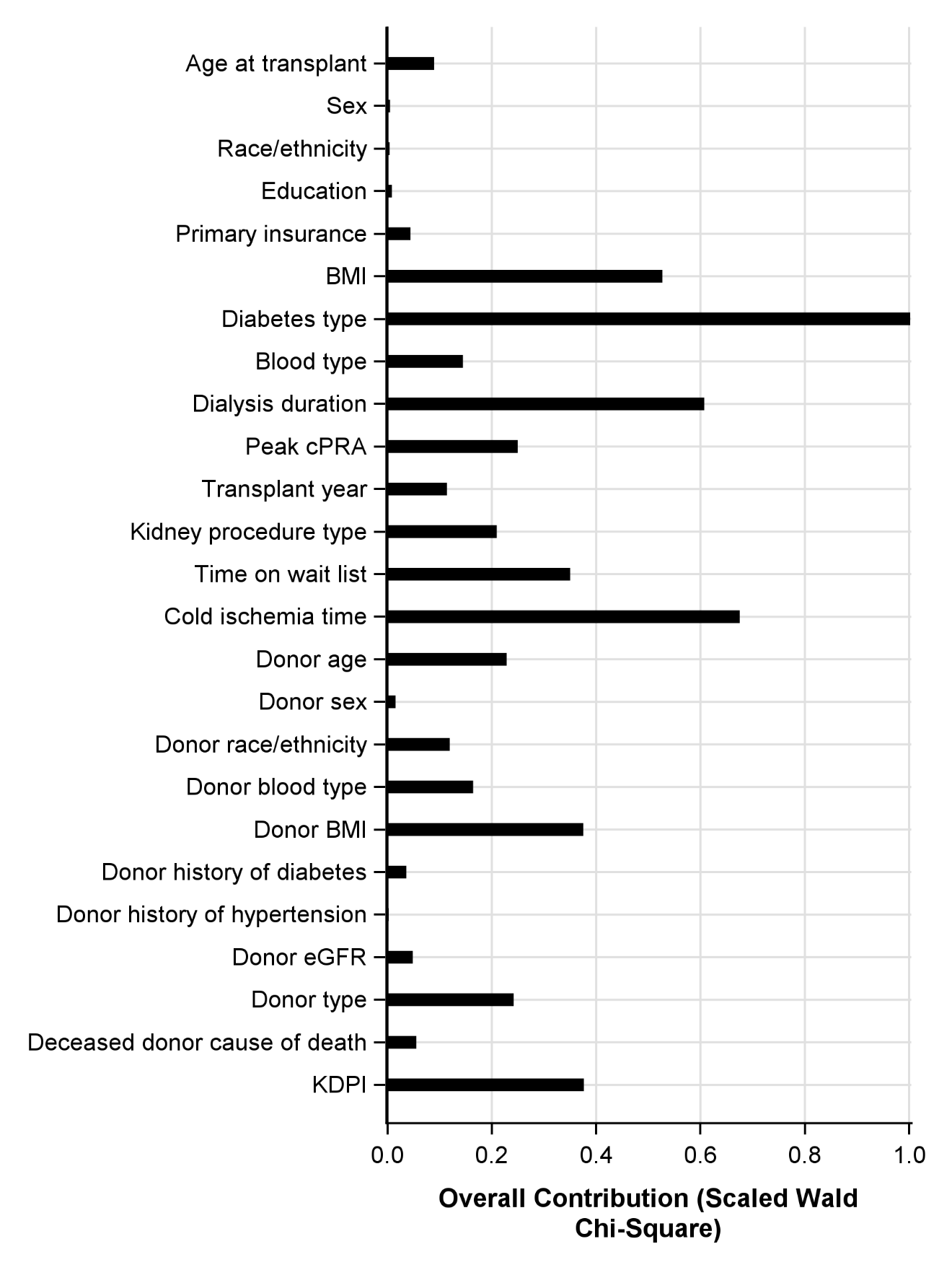


**Supplemental Figure 4. Unadjusted Cumulative Pancreas Graft Failure Rates by Age Group**


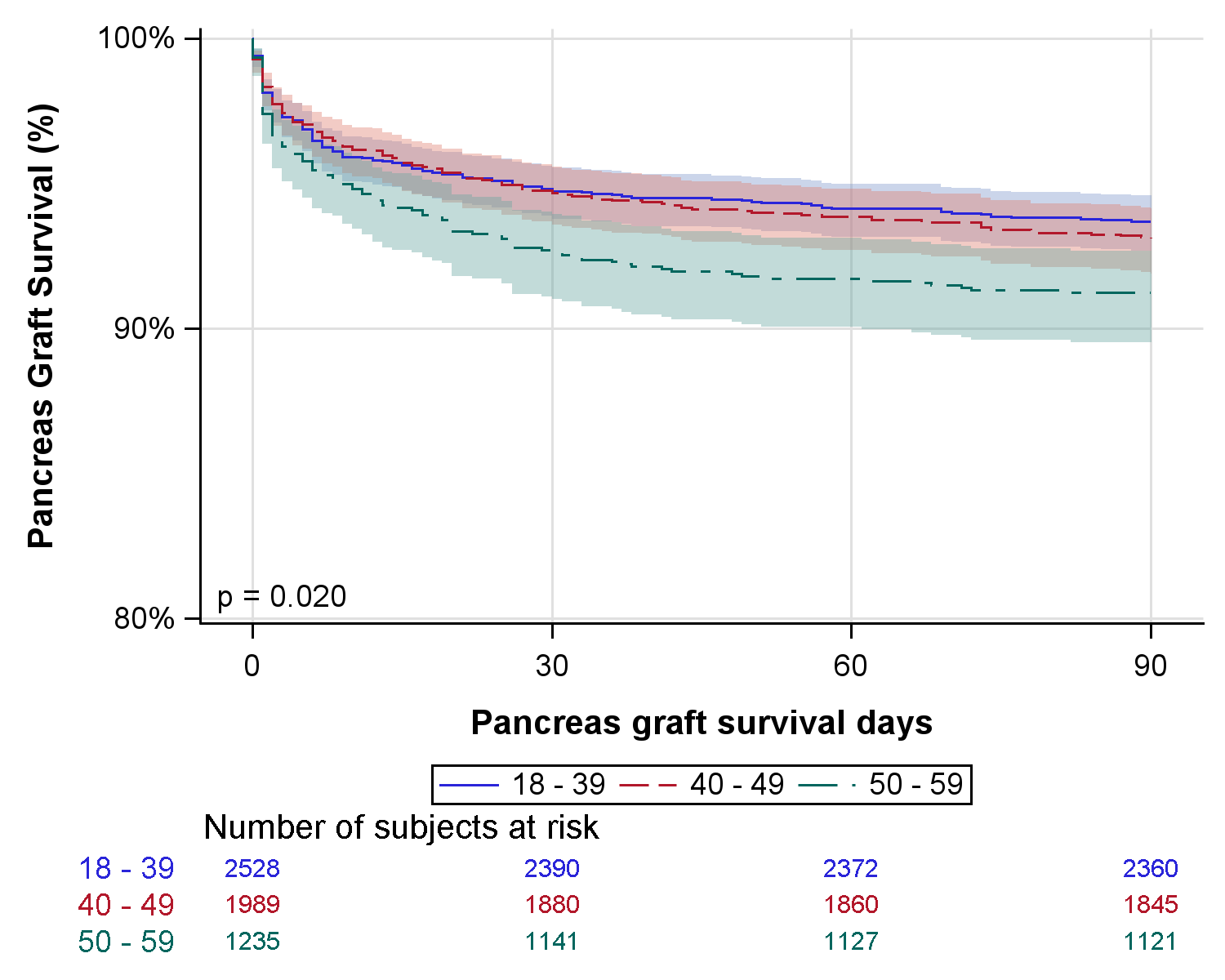


**Supplemental Table 1. The association of DDKT vs. SPKT with patient mortality and kidney graft failure, A sensitivity analysis of patients with Type 1 diabetes**

| **Model** | **DDKT vs. SPKT HR (95% CI)** | |
| --- | --- | --- |
|  | **Kidney Graft Failure** | **Patient Mortality** |
| **Subset: Type 1 Diabetes (n = 6,512)** | | |
| Unadjusted | 1.70 (1.51, 1.93) | 2.13 (1.84, 2.46) |
| Covariate-adjusted* | 1.03 (0.84, 1.27) | 1.17 (0.92, 1.49) |
| **Subset: Type 1 Diabetes with BMI < 30 (n = 5,050)** | | |
| Unadjusted | 1.67 (1.43, 1.95) | 2.20 (1.84, 2.62) |
| Covariate-adjusted* | 1.13 (0.89, 1.45) | 1.37 (1.04, 1.81) |

*Adjusted for recipient sex, age, race/ethnicity, education, insurance, blood type, BMI, dialysis months, cPRA, transplant year, left vs right kidney transplant, months on wait list, CIT, donor sex, donor age, and KDPI

CI: confidence interval; DDKT: deceased donor kidney transplant; HR: hazard ratio; SPKT: simultaneous pancreas-kidney transplant

**Supplemental Table 2. The association of SPKT vs. DDKT with treated acute rejection and hospital readmission, A sensitivity analysis of patients with Type 1 diabetes**

| **Model** | **SPKT vs. DDKT OR (95% CI)** | |
| --- | --- | --- |
|  | **Acute Treated Rejection**  **Within 1 Year of Transplant^1^** | **Hospital Readmission**  **Within 1 Year of Transplant^1^** |
|  | **Subset: Type 1** | |
| Num. of observations | 5,658 | 5,636 |
| Unadjusted | 2.29 (1.80, 2.91) | 1.29 (1.15, 1.45) |
| Covariate-adjusted^2^ | 2.34 (1.61, 3.39) | 1.73 (1.43, 2.10) |
|  | **Subset: Type 1 with BMI < 30** | |
| Num. of observations | 4,437 | 4,414 |
| Unadjusted | 2.52 (1.82, 3.50) | 1.19 (1.02, 1.38) |
| Covariate-adjusted^2^ | 2.08 (1.33, 3.25) | 1.62 (1.28, 2.04) |

^1^ Restricted to the subset of subjects with the one-year post-transplant follow-up form who had not experienced graft loss within the first post-transplant year.

^2^ Adjusted for sex, age, race/ethnicity, education, insurance, blood type, BMI, dialysis months, cPRA, diabetes type, transplant year, kidney transplant type, months on wait list, CIT, donor sex, donor age, and KDPI

CI: confidence interval; DDKT: deceased donor kidney transplant; OR: odds ratio; SPKT: simultaneous pancreas-kidney transplant
